# Comparison of Impacts of Essential Oils, Green Tea Powder, Betaine, Probiotics, and Other Dietary Supplements on Growth and Well-Being of Heat-Stressed White Pekin Ducks

**DOI:** 10.3390/ani15233382

**Published:** 2025-11-22

**Authors:** Jill R. Domel, Eric B. Sobotik, Gabrielle M. House, Gregory S. Archer

**Affiliations:** Department of Poultry Science, Texas A&M University, College Station, TX 77845, USA; jill.domel@ag.tamu.edu (J.R.D.);

**Keywords:** heat stress, ducks, probiotics, essential oils, green tea, betaine, intestinal health

## Abstract

Three experiments were conducted to compare different dietary supplements fed to mixed-run White Pekin ducks (*n* = 48 birds/treatment) reared for 35 d and exposed to ambient temperatures from d 0 to 35 or heat stress (32 °C–35 °C) for 12 h/d on d 20–35. The basal diet (CON) was supplemented with 0.5 kg/MT seaweed extract (SE), 2.0 kg/MT betaine (BET), or 1.25 kg/MT yeast fermentate (YF) in Experiment 1; with 0.25 kg/MT mannanoligosaccharide (MOS), 0.5 kg/MT green tea powder (GT), or 0.55 kg/MT Poultry Star synbiotic (PS) in Experiment 2; or with 0.09 kg/MT Essential Oil #1 (EO1), 0.5 kg/MT Essential Oil #2 (EO2), or 0.5 kg/MT Microsaf probiotic (MS) in Experiment 3. Heat stress reduced body weight and feed consumption (Exp. 1–3) and negatively affected measures of stress (Exp. 1 and 2) and at least one measure of intestinal health (Exp. 1–3). Intestinal morphology was improved in birds that were fed EO1, EO2, and MS, and some measures of stress susceptibility were improved in birds that were fed SE, YF, MOS, GT, BET, or PS. Essential oil supplementation may be best used in combination with another of the supplements tested to optimize health and welfare in Pekin ducks. Further research could clarify which combinations are ideal for supporting both intestinal health and stress susceptibility in ducks.

## Simple Summary

Ducks are commonly reared for their meat in parts of the world which are prone to extreme heat, which can negatively affect their growth and well-being. However, some dietary supplements may counteract such effects. This study aimed to compare several different types of supplements, including prebiotics, probiotics, synbiotics (prebiotic and probiotic combination), seaweed extract, green tea extract, betaine, and essential oil blends and their impacts on measures of growth, health, and stress in White Pekin ducks exposed to either ambient temperature or heat stress. Although body weight gain and feed efficiency were not affected by any of the tested supplements, adding essential oils to the diet improved measures of intestinal health, whereas all other tested supplements improved some measures of stress. Feeding a combination of essential oils and another of the supplements tested in this study could improve both intestinal health and well-being in ducks during the growth period.

## 1. Introduction

Heat stress has become an increasingly pressing concern in livestock production, with the risk of extreme heat projected to increase in tropical and temperate zones over the next 75 years [1]. Heat stress occurs when an animal is exposed to environmental conditions which exceed its ability to dissipate heat using behavioral or physiological adaptations. Heat stress can manifest a range of negative impacts on poultry health and well-being. For example, previous research has shown that production measures and breast meat quality declined when ducks were exposed to upper critical temperatures ranging from 25.5 °C to 27.4 °C [2]. Ducks exposed to chronic heat stress experience reduced antioxidant capacity [3,4], leading to inflammatory injury to tissues such as the liver [3]. Heat-induced oxidative stress has also been shown to affect intestinal morphology [5] and microflora, resulting in poor growth performance [4]. Ducks exposed to cyclic heat stress before slaughter demonstrated reduced meat quality in terms of both nutritional content and protein functionality [6], as well as increased abdominal fat and reduced skeletal muscle integrity [7].

Various dietary supplements have been evaluated for their potential in mitigating the negative effects of heat stress and thereby improving duck well-being and production performance. For example, the probiotic mannanoligosaccharide (MOS) has been shown to prevent certain types of pathogenic bacteria from colonizing the surface of poultry intestines through adsorption without negatively affecting nonpathogenic microflora [8]. At levels of 1 kg/ton, MOS supplementation can also reduce feed consumption and feed conversion ratio (FCR) in meat ducks [9]. On the other hand, the fermentation product of *Saccharomyces cerevisiae* can be used as a prebiotic: yeast fermentate contains yeast cells, yeast cell wall fragments, metabolites, and media on which the yeast was grown [10]. Yeast fermentate supplementation has been shown to mitigate heat stress-induced changes in intestinal morphology in Pekin ducks [5]. Alternatively, synbiotics, which contain synergistic combinations of prebiotics and probiotics, have also been tested. Broilers supplemented with synbiotic have yielded higher body weights and improved feed conversion ratio [11,12,13], as well as increased carcass yield [11] than those fed a control diet. Others have found that while synbiotic supplementation has different effects on growth performance in heat-stressed broilers depending on the stage of development, synbiotic-supplemented birds have improved welfare measures [14]. Adding a synbiotic to the diet can also improve intestinal histomorphology in broilers [11] and ducks [13], and more specifically, ameliorate the negative effects of heat stress on these measures [15].

Other feed additives used to relieve the effects of heat stress and improve growth in poultry include plant extracts. For instance, *Ascophyllum nodosum* seaweed extract (SE) contains bioactive compounds which may have antioxidant and antimicrobial effects, including alginic acid, phenolic compounds, phlorotannins, and protein, as well as vitamins and minerals [16,17]. Heat-stressed broiler chickens supplemented with SE have shown reduced plasma corticosterone (CORT) and heterophil/lymphocyte (H/L) ratios, measures of long-term stress, as well as reduced levels of heat shock protein 70 [18]. Some researchers have also assessed the potential of supplementing green tea (*Camellia sinensis,* GT) powder and extracts in broiler and layer diets, although there is little information on its use in ducks. For example, Zhang et al. [19] found that L-theanine, an amino acid in GT, has positive effects on intestinal morphology and growth performance in ducks when fed at 600 to 900 mg/kg. Other researchers have analyzed the effect of epigallocatechin gallate (EGCG), another molecule found in GT extract, in laying ducks and found that supplementing 300 mg/kg in the diet improved egg production and antioxidant capacity during heat stress [20].

Alternatively, the addition of certain essential oils to poultry diets has been investigated in terms of growth performance and intestinal health, and essential oil supplementation has been shown to improve antioxidant capacity [21,22]. Some researchers have also found that essential oils affected intestinal health, including modulating ceca microbiota [20,22,23] and improving gene expression related to intestinal barrier function [21,24]. Essential oil supplementation has also shown some improvement in intestinal morphology as well as improved body weight gain and feed conversion ratio [21,24,25], although there is some variability in these findings [22]. It has been suggested that the varying effects of essential oils on growth performance and intestinal measures indicate a dose-dependent relationship [26].

Finally, betaine has been investigated for its effectiveness in mitigating the impact of heat stress on poultry growth and well-being. Betaine is a derivative of the amino acid glycine and can donate methyl groups for the synthesis of other substances, including methionine, thereby playing a role in protein synthesis and energy metabolism [27]. When supplemented in the diet, betaine improves body weight gain and FCR in heat-stressed [28,29] and non-heat-stressed ducks [30]. In addition, betaine supplementation increases propionic acid and acetic acid, two short-chain fatty acids, in the cecum of heat-stressed ducks [28]. Furthermore, betaine is a zwitterionic molecule and is known to regulate osmotic pressure in a variety of vertebrates and invertebrates [27]. Given its role in metabolism and osmotic regulation, betaine may reduce intestinal cell damage during heat stress [31], thus improving growth performance. Its role in osmoregulation and prevention of cellular damage is further evidenced by reduced drip loss in duck carcasses [30].

Although many studies have measured the effectiveness of these dietary supplements on poultry health and performance, few have compared multiple additives in a single experiment. In addition, more information is needed to determine the usefulness of these additives in ducks exposed to chronic heat stress. Therefore, the objective of the experiments presented is to compare the effects of the supplementation of prebiotic, probiotic, synbiotic, GT extract, two proprietary essential oil blends, and betaine on stress susceptibility, intestinal histomorphology, and growth performance of White Pekin ducks exposed to cyclic heat stress during the latter half of the rearing period.

## 2. Materials and Methods

### 2.1. Animals and Husbandry

All procedures were carried out in accordance with the guidelines established by the Texas A&M Institutional Animal Care and Use Committee (AUP# 2017-0427) and the Guide for the Care and Use of Agricultural Animals in Research and Teaching [32]. Ducks were housed at the Texas A&M University Poultry Science Teaching, Research, and Extension Center, and all diets were mixed on site.

There were 48 ducks in each treatment. Heat-stressed and non-heat-stressed treatments were separated into two identical rooms to control environmental conditions. Heat-stressed treatments were assigned to one room and non-heat-stressed treatments assigned to a separate room, and the assigned room was consistent for both experiments. Pens measured 0.91 × 1.83 m and were lined with 3–5 cm of fresh pine shavings. Each pen was equipped with one tube feeder and one drinker consisting of an 18.93 L plastic bucket with four nipples on the bottom. There were four pens for each treatment in each experiment, with 12 ducks/pen. Pens were assigned to treatments using a randomized complete block design, with four blocks in each room. Ducks were fed a crumbled starter diet on d 0–14 and a pelleted grower diet on d 15–35. The ingredient composition of the diet is shown in Table 1. A pellet binder (0.91 kg/MT calcium lignosulfonate from Producer’s Co-Op, Bryan, TX, USA) was included in the basal grower diet to maintain pellet quality. Ducks had ad libitum access to feed and water for the duration of the trial, and feeders and waterers were raised as ducks grew.

Room temperature was maintained at 31 °C from d 0–3, then reduced by 2 °C every other day for the next week until an ambient temperature of 21–24 °C was reached. Non-heat-stressed treatments were maintained at this ambient temperature for d 0–35 for all three experiments. Heat-stressed treatments were maintained at ambient temperatures for d 0–19, then exposed to elevated temperatures of 32–35 °C for 12 h/d on d 20–35 (2 h after lights turned on until 2 h before lights turned off). All treatments were exposed to a 21 L:3D photoperiod on d 0–3, followed by a 16 L:8D photoperiod on d 4–35.

### 2.2. Dietary Additives

The following were supplemented to the basal diet d 0–35 for each treatment for Experiment 1: 0.5 kg/MT seaweed extract (Tasco, Dartmouth, NS, Canada) for the SE treatment; 2.0 kg/MT betaine (The Archer–Daniels–Midland Company, Chicago, IL, USA) for the BET treatment; 1.25 kg/MT yeast fermentate (Diamond V, Cedar Rapids, IA, USA) for the YF treatment.

For Experiment 2, the following were supplemented to the basal diet d 0–35 for each treatment: 0.25 kg/MT mannanoligosaccharide (Safmannan; Phileo by Lesaffre, Marcq-en-baroeul, Nord-Pas-de-Calais, France) for the MOS treatment; 0.5 kg/MT GT extract powder (Hard Eight Nutrition LLC, Henderson, NV, USA) for the GT treatment; 0.55 kg/MT Poultry Star symbiotic (DSM, Heerlen, The Netherlands) for the PS treatment.

For Experiment 3, the following were supplemented to the basal diet d 0–35 for each treatment: 0.09 kg/MT essential oil blend #1 (Biomin, Inzersdorf-Getzersdorf, Austria) for the EO1 treatment; 0.5 kg/MT probiotic (Microsaf; Phileo by Lesaffre, Marcq-en-baroeul, Nord-Pas-de-Calais, France) for the MS treatment; 0.5 kg/MT essential oil blend #2 (Biomin, Inzersdorf–Getzersdorf, Austria) for the EO2 treatment.

### 2.3. Production Measures

All pens were checked for mortality daily. Any mortalities were promptly removed and the pen number was recorded for each incident; this information was then used to calculate total mortality from d 0 to 35 for each treatment. Feed was weighed each time it was added to the feeder for each pen, and the weight of remaining feed was recorded before transitioning from the starter to the grower diet and on d 35 to calculate total feed consumption for d 0 to 35. On d 0 and 35, all ducks in each pen were weighed using a rolling scale (UFM-F120, UWE Scales, Cape Town, South Africa) to calculate total body weight gain for each pen. Then, feed conversion ratio (FCR) for d 0 to 35 was calculated by dividing the total feed intake for each pen by the total body weight gain for each pen, corrected for mortality.

### 2.4. Stress Susceptibility

On d 35, 1–2 mL of blood was collected from the brachial wing vein from each of 20 randomly selected ducks per treatment. Whole blood from each duck was transferred to a heparin and lithium gel separation vacutainer (367884, BD Medical, Franklin Lakes, NJ, USA). One drop of whole blood from each duck was also used to create a blood smear to determine heterophil/lymphocyte (H/L) ratio. Dry blood smear slides were stained with a neat stain hematology stain kit (Cat. #25034, Poly Sciences, Inc., Warrington, PA, USA). Heterophil/lymphocyte ratio was determined by individually counting heterophils and lymphocytes up to a total of 100 cells per slide at 40× magnification using an oil immersion lens under microscopy (89404-886, VWR International, Radnor, PA, USA). Vacutainers containing whole blood were inverted 2 to 3 times and stored in an ice bath until remaining blood samples were collected. Vacutainers were then centrifuged (Centrifuge 5804, Eppendorf, Hamburg, Germany) at 4000 RPM for 15 min and plasma was transferred to a microcentrifuge tube. Plasma samples were stored at −20 °C, then thawed overnight at 4 °C for analysis. Plasma CORT concentration was obtained using a 96-well commercial ELISA kit (ADI-901-097, Enzo Life Sciences, Inc., Farmingdale, NY, USA). Absorbance was read at 450 nm using a microplate absorbance reader (Tecan Sunrise, Tecan Trading AG, Männedorf, Switzerland) and analyzed using the Magellan Tracker software program (version 7.2).

### 2.5. Intestinal Histomophology

Ten ducks were randomly selected from each treatment on d 35 and euthanized via captive bolt. Then, a 1 cm long section of ileum was collected from the mid-way point between Meckel’s diverticulum and the ileocecal junction. Ileum samples were gently rinsed with phosphate-buffered saline and then stored in 10 mL of 10% neutral buffered formalin at room temperature. Samples were sent to Histo-Scientific Research Laboratories (Mt. Jackson, VA, USA) for processing and staining. Samples were stained with Periodic Acid-Schiff and Alcian Blue. Mounted and stained ileum sections were photographed at 40× magnification using a Nikon Eclipse Ci-L microscope (Nikon Corporation, Tokyo, Japan). The accompanying Elements software package (version 5.02) was used to measure villus height and crypt depth in μm from six villi per sample. Villus height was divided by crypt depth to determine villus/crypt (V/C) ratio.

### 2.6. Statistical Analysis

Data was analyzed as a 2 × 3 factorial design using the General Linear Model in Minitab 17.1.0 (Minitab, Inc., State College, PA, USA) to test the main effects of heat and diet and their interaction. Pairwise comparisons were also made using Fisher’s Least Significant Differences. A significant difference was defined as *p* < 0.05.

## 3. Results

### 3.1. Experiment 1

#### 3.1.1. Production Measures

Feed consumption, FCR, d 35 body weight, and mortality for Experiment 1 are shown in Table 2. There was a main effect of heat stress on d 35 body weight (*p* < 0.001) and feed consumption (*p* < 0.001), both of which were lower in heat-stressed ducks compared to those reared under ambient environmental temperature. There was no main effect of heat (*p* > 0.05) or diet (*p* > 0.05), or heat x diet interaction (*p* > 0.05) on FCR or mortality.

#### 3.1.2. Stress Susceptibility and Intestinal Histomorphology

Data for Experiment 1 plasma CORT, H/L ratio, villus length, crypt depth, and V/C ratio are presented in Table 3. There was a main effect of heat stress (*p* = 0.001) on plasma CORT, which was higher in heat-stressed ducks than in those reared under ambient environmental temperature. Main effects of diet (*p* = 0.004) and heat stress (*p* = 0.001) were observed for H/L ratio, but there was no interaction effect (*p* > 0.05) observed. H/L ratio was higher in heat-stressed ducks than non-heat-stressed ducks; H/L ratio was higher in ducks fed the CON diet than those supplemented with SE, BET, or YF. The main effect of heat stress (*p* = 0.024) was also observed for villus length. Heat-stressed ducks had lower villus length than those reared under ambient temperatures.

### 3.2. Experiment 2

#### 3.2.1. Production Measures

Data for Experiment 2 feed consumption, FCR, d 35 body weight, and mortality are presented in Table 4. Heat stress had a significant effect on d 35 body weight (*p* < 0.001) and feed consumption (*p* = 0.005), both of which were lower in heat-stressed ducks than those reared under ambient temperature. We did not observe the main effect of diet (*p* > 0.05) nor a heat x diet interaction (*p* > 0.05) for any of the production measures in Experiment 2.

#### 3.2.2. Stress Susceptibility and Intestinal Histomorphology

Data for Experiment 2 plasma CORT, H/L ratio, villus length, crypt depth, and V/C ratio are presented in Table 5. There was a main effect of heat on plasma CORT (*p* = 0.003) and crypt depth (*p* = 0.016). Heat-stressed ducks had higher plasma CORT and lower crypt depth than those reared under ambient temperatures. There was a significant effect of heat (*p* = 0.026) as well as a heat x diet interaction (*p* = 0.01) on H/L ratio. The PS + H treatment had a higher H/L ratio than PS + A. Furthermore, H/L ratio of CON + H was higher than MOS + A and PS + A. We did not observe the main effect of diet (*p* > 0.05) for any of these measures in Experiment 2.

### 3.3. Experiment 3

#### 3.3.1. Production Measures

Data for Experiment 3 feed consumption, FCR, d 35 body weight, and mortality are presented in Table 6. There was a main effect of heat stress on d 35 body weight (*p* < 0.001) and on d 0–35 feed consumption (*p* = 0.005) both of which were lower in heat-stressed ducks than those reared under ambient temperature. We did not observe the main effect of diet (*p* > 0.05) nor a heat x diet interaction (*p* > 0.05) for any of the production measures in Experiment 3.

#### 3.3.2. Stress Susceptibility and Intestinal Histomorphology

Data for Experiment 3 plasma CORT, H/L ratio, villus length, crypt depth, and V/C ratio are presented in Table 7. There was a main effect of heat stress on villus length (*p* = 0.018), which was lower in heat-stressed ducks than those reared under ambient temperature. There was a main effect of diet on crypt depth (*p* = 0.034) and V/C ratio (*p* = 0.032). Crypt depth was higher, and V/C ratio was lower in ducks fed the CON diet than those supplemented with EO1 or EO2. There was no main effect of heat (*p* > 0.05) or diet (*p* > 0.05) observed for H/L ratio. However, there was a heat x diet interaction (*p* = 0.012) effect on H/L ratio, which was higher in EO1 + H compared to CON + H, EO2 + H, and EO1 + A.

## 4. Discussion

Exposure to heat stress decreased feed consumption and body weight in all three experiments, which reflects findings from other researchers [3,4]. In addition, heat-stressed ducks exhibited higher plasma CORT and H/L ratios, indicators of stress in poultry [32], in Experiments 1 and 2. This is consistent with previous research [3,33], and suggests that the use of cyclic heat stress for 12 h/d on d 21–35 of the rearing period was successful in inducing stress. It is surprising, however, that there was not a main effect of heat on feed conversion ratio, as exposure to heat stress has had a negative impact on feed efficiency in ducks [4], particularly after d 21 of the rearing period [28]. On the other hand, heat-stressed ducks had reduced villus length than those reared under ambient temperature in Experiments 1 and 3 as well as reduced crypt depth in Experiment 2. Enterocytes on the luminal surface of the villus migrate toward the tip of the villus and eventually slough off, and new cells arise primarily from proliferation of stem cells in the crypt [34]. Exposure to heat stress increases the rate of cell turnover [35], making intestinal morphology measurements such as villus length, crypt dept, and villus/crypt ratio reliable indicators of intestinal health. Other research has shown that ducks exposed to heat stress for 6, 8, or 12 h/d had shorter villi and reduced V/C ratio [4,5,35]. Liu et al. [35] found that ducks exposed to heat stress for 3, 6, or 12 h/d had higher crypt depth, whereas others did not find an effect of heat stress for 8 h/d on crypt depth [4,5]. Exposing White Pekin ducks to heat stress for 12 h/d appears to have been effective in altering intestinal histomorphology in this study.

There was no main effect of diet on feed consumption, FCR, body weight, or mortality in Experiment 1, 2, or 3. Neither was there a heat × diet interaction observed for these measures in Experiment 3. In addition, there was no main effect of diet on intestinal morphology in Experiment 1 or 2. Pairwise comparisons showed that CON + H ducks had higher plasma CORT and H/L ratio than all other treatments. However, there was a main effect of diet on H/L ratio, which was improved in ducks fed SE, BET, and YF compared to those fed the CON diet. In terms of growth and intestinal health, all non-heat-stressed treatments had higher body weight than their heat-stressed counterparts except for YF-supplemented ducks, and ducks in the SE + A treatment had longer villi than those in BET + A and CON + H. Therefore, although SE, BET, and YF supplementation improved H/L ratio, villus ratio was reduced with BET-supplemented ducks but improved in those fed SE. Furthermore, SE and BET supplementation increased feed consumption in non-heat-stressed ducks without improving feed conversion ratio or body weight gain. These findings agree with previous research showing that YF-supplemented broilers exposed to heat stress or other acute stress had lower plasma CORT and H/L [36]. In addition, Klasing et al. [37] showed that betaine did not affect duodenum villus height or crypt depth in broiler chicks, while others found no effect of supplementing betaine in the feed or drinking water on production performance or carcass traits in ducks exposed to cyclic heat stress [38]. To maximize its impact on stress and well-being, it has been suggested that BET be supplemented via the feed or drinking water before heat stress is anticipated to occur, rather than limiting supplementation to the period when birds are exposed to heat stress [31]. However, the results of this study indicate no such benefit in terms of growth performance or intestinal health. Taken together, adding SE, YF, or BET to the feed improves some measures of stress in Pekin ducks; however, SE and YF supplementation showed more promise than BET in maintaining intestinal health in ducks reared under ambient temperature.

In Experiment 2, pairwise comparisons showed that PS + A ducks had lower H/L ratio than PS + H. In addition, both PS + A and MOS + A had lower H/L ratio than CON + A. Mohammed et al. [39] found that heat-stressed broilers had lower H/L ratio with increasing synbiotic levels in the diet, although synbiotic supplementation did not appear to influence plasma CORT in the same study. In this experiment, however, ducks fed the PS or CON diet had lower plasma CORT than their heat-stressed counterpart. Others have found that feeding a synbiotic reduced production of heat shock proteins as well as improved intestinal morphology in heat-stressed broilers [40]. Indeed, feeding a synbiotic was found to be more effective in ameliorating the negative effects of high stocking density on intestinal histomorphology compared to feeding MOS alone [41]. However, PS + H had lower feed consumption than CON + A and MOS + A, and although another synbiotic tested in broilers showed improvements in crypt depth, villus height, and V/C ratio [42], no differences in intestinal morphology were observed in this study. Neither was there an effect of diet on body weight or FCR. Thus, adding PS to the diet improved measures of stress in ducks exposed to ambient temperature without affecting intestinal morphology or growth performance.

Among heat-stressed treatments in Experiment 2, ducks supplemented with MOS and GT had lower plasma CORT than those fed the CON diet, as well as lower H/L ratio than ducks fed PS. This agrees with previous findings of GT extract supplementation reducing plasma CORT in broilers [43], although the results of this study differed in that GT supplementation did not appear to influence growth performance, possibly due to differences in the concentration of polyphenols obtained from different methods of GT preparation [44]. This reduction in stress may be attributed to the antioxidant effects of EGCG found in green tea extract [42]. Furthermore, others have found that MOS supplementation at the same dose used in this study mitigated the effects of heat stress on measures of intestinal barrier function, oxidative stress, and growth performance in broilers [45]. Ducks fed MOS did not show such improvements in growth performance or intestinal morphology, which could be due to differences in physiology between ducks and broiler chickens. However, ducks fed MOS still benefited from some reduction in stress measures, which may be related to improvements in oxidative status [45]. Therefore, feeding PS, GT, and MOS improved some measures of stress in Pekin ducks in this study without affecting intestinal morphology or growth performance. Further research could clarify the role of GT, MOS, and PS supplementation in reducing stress through the assessment of other health indicators such as antioxidant status during heat stress exposure.

Pairwise comparisons in Experiment 3 showed that ducks fed EO1 and EO2, regardless of exposure to heat stress, had higher V/C ratio than ducks fed the control diet. In addition, MS + H ducks had longer villi compared to CON + H; otherwise, MS supplemented ducks did not show improvements in growth performance, crypt depth, or villus/crypt ratio. Thus, supplementing either of the proprietary essential oils tested in this study supported intestinal health in both ambient and high environmental temperatures, and supplementing the MS probiotic improved villus length during heat stress but not ambient temperature. Oregano oil and other essential oils have shown a positive effect on cecal microbiota [21,23], antioxidant capacity [23], and mucosal barrier integrity [24]. Dietary supplementation of essential oils has shown mixed results with respect to intestinal morphology in non-heat-stressed ducks. One study showed a negative effect of oregano oil on villus length and V/C ratio when supplemented in the drinking water [25]. However, Lixuan et al. [26] suggested that the effect of supplementing essential oils in drinking water on intestinal morphology in ducks is dose dependent. Others have found that adding essential oils to the feed improves these same parameters [21], particularly when stabilized through encapsulation [24]. Neither of the essential oil supplements tested in this study improved growth performance, which aligns with others’ findings [22,23]; nor did ducks fed either the EO1 or EO2 diet show reduced measures of stress. However, we did not observe a negative effect of essential oil supplementation on growth performance in ducks reared under heat stress or ambient conditions. Taken together, the improvements in intestinal histomorphology in EO-supplemented ducks in this study may be the result of reduced intestinal cell turnover attributed to enhanced intestinal barrier function and microflora.

## 5. Conclusions

This study aimed to elucidate which of several types of supplements commonly used in poultry production would have a greater impact on growth performance and stress susceptibility in heat-stressed Pekin ducks. We found that although performance indicators such as mortality, body weight, and FCR were not significantly affected with supplementation of any of the products tested, both essential oil blends and MS probiotic improved measures of intestinal health. Ducks supplemented with YF, SE, BET, GT, MOS, or PS showed some improvement in stress susceptibility, although PS, SE, BET showed mixed results in terms of impact on feed consumption and intestinal health. It is possible that some of the improvements in stress susceptibility observed in this study are related to improved antioxidant status and gut microflora. Therefore, it is suggested to include YF (1.25 kg/MT), MOS (0.25 kg/MT), or GT (0.5 kg/MT) in the ratios of the doses tested in this study to reduce susceptibility to long-term stress without negatively affecting growth performance during the entire rearing period, whether or not ducks may be exposed to high environmental temperatures. In addition, feeding Pekin ducks a combination of an essential oil blend and another supplement tested in this study may optimize both intestinal health and stress susceptibility in Pekin ducks reared under cyclic heat stress conditions. Further research could clarify which combinations yield optimal results.

## Figures and Tables

**Table 1:** Ingredient composition of the basal diets for the starter (fed d 0–14) and grower (fed d 15–35) diet phases ^1^.

Ingredient	Starter (%; d 0–14)	Grower (%; d 15–35)
Corn	43.37	55.23
Soybean meal	39.70	27.28
Soy Oil	5.91	7.90
Wheat Middlings	6.02	6.01
DL-Methionine, 98%	0.36	0.27
L-Lysine	0.01	0.08
Limestone	2.67	1.18
Monocalcium phosphate	1.25	1.32
Salt	0.42	0.42
Trace Mineral Mix ^2^	0.05	0.05
Vitamin Premix ^3^	0.25	0.25

^1^ Pellet binder (calcium lignosulfonate) was added to the grower diet at a rate of 2.72 g/kg feed. ^2^ Trace mineral premix added at this rate yields the following per kilogram: 13.33 g manganese, 13.33 g zinc, 13.33 g iron, 1.56 g copper, 0.09 g iodine, a minimum of 1.39 g calcium and a maximum of 1.93 g calcium. Calcium carbonate was used as a carrier. ^3^ Vitamin premix added at this rate yields the following per kilogram: 36,741.67 IU vitamin A, 12,860 IU vitamin D3, 151.67 IU vitamin E, 435.17 mg choline, 153 mg niacin, 67.33 mg D-pantothenic acid, 23.83 mg pyridoxine, 19.83 mg riboflavin, 9.78 mg thiamin, 5.83 mg folic acid, 1.83 mg biotin, and 0.07 mg vitamin B12.

**Table 2:** Experiment 1 production outcomes of White Pekin ducks fed various dietary additives and reared under heat stress or ambient temperatures from d 0 to 35.

Treatment *	Feed Consumption (kg; d 0–35)	Feed Conversion Ratio (d 0–35)	Body Weight (kg; d 35)	Mortality (%; d 0–35)
CON + H	122.45 ^de^	1.58 ^ab^	2.78 ^c^	4.17
SE + H	126.48 ^cd^	1.59 ^ab^	2.84 ^bc^	2.08
BET + H	119.05 ^e^	1.58 ^ab^	2.71 ^c^	6.25
YF + H	127.75 ^cd^	1.63 ^a^	2.83 ^bc^	0.00
CON + A	131.47 ^bc^	1.54 ^b^	3.07 ^a^	4.17
SE + A	141.61 ^a^	1.61 ^ab^	3.16 ^a^	0.00
BET + A	138.95 ^a^	1.60 ^ab^	3.14 ^a^	4.17
YF + A	135.55 ^ab^	1.61 ^ab^	3.00 ^ab^	6.25
Heat × Diet *p*-value	0.088	0.608	0.307	0.345
Main Effect Heat				
Heat Stress	123.93 ^b^	1.60	2.79 ^b^	3.65
Ambient	136.89 ^a^	1.59	3.10 ^a^	3.13
*p*-value	<0.001	0.761	<0.001	0.778
Main Effect Diet				
CON	126.96	1.56	2.93	4.17
SE	134.04	1.60	3.00	1.04
BET	129.00	1.59	2.93	5.21
YF	131.65	1.62	2.91	3.13
*p*-value	0.053	0.312	0.561	0.434
SEM	1.55	0.01	0.04	0.91

^a–e^ Means in the same column with different superscripts indicate a significant difference at *p* < 0.05. * Ducks were fed either a basal diet (CON) or supplemented with 0.5 kg/MT seaweed extract (SE), 2.0 kg/MT betaine (BET), or 1.25 kg/MT yeast fermentate (YF) and exposed to ambient temperatures d 0–35 (CON + A, SE + A, BET + A, YF + A) or subjected to heat stress for 12 h/d on d 21–35 (CON + H, SE + H, BET + H, YF + H).

**Table 3:** Experiment 1 stress susceptibility measures and ileal histomorphology of White Pekin ducks fed various dietary additives and reared under heat stress or ambient temperatures from d 0 to 35.

Treatment *	Plasma CORT (pg/mL; d 35)	Heterophil/ Lymphocyte Ratio (d 35)	Villus Length (µm)	Crypt Depth (µm)	V/C Ratio
CON + H	11,956.2 ^a^	0.55 ^a^	506.42 ^c^	143.68	4.02
SE + H	8401.3 ^b^	0.39 ^b^	562.38 ^abc^	137.08	4.48
BET + H	7939.3 ^b^	0.36 ^b^	556.46 ^abc^	138.26	4.47
YF + H	8053.1 ^b^	0.38 ^b^	585.99 ^abc^	141.26	4.56
CON + A	6537.1 ^b^	0.37 ^b^	601.60 ^ab^	144.30	4.52
SE + A	6373.7 ^b^	0.31 ^b^	643.80 ^a^	148.59	4.98
BET + A	6323.9 ^b^	0.33 ^b^	551.08 ^bc^	162.45	4.19
YF + A	6883.9 ^b^	0.32 ^b^	620.57 ^ab^	128.84	5.24
Heat × Diet *p*-value	0.182	0.248	0.373	0.409	0.621
Main Effect Heat					
Heat Stress	9087.47 ^a^	0.42 ^a^	552.81 ^b^	140.07	4.39
Ambient	6529.63 ^b^	0.33 ^b^	604.26 ^a^	146.04	4.73
*p*-value	0.001	0.001	0.024	0.455	0.219
Main Effect Diet					
CON	9246.63	0.46 ^a^	554.01	143.99	4.27
SE	7387.50	0.35 ^b^	603.09	142.83	4.73
BET	7131.56	0.34 ^b^	553.77	150.36	4.33
YF	7468.50	0.35 ^b^	603.28	135.05	4.90
*p*-value	0.182	0.004	0.190	0.596	0.315
SEM	397.67	0.01	11.53	3.90	0.14

^a,b,c^ Means in the same column with different superscripts indicate a significant difference at *p* < 0.05. * Ducks were fed either a basal diet (CON) or supplemented with 0.5 kg/MT seaweed extract (SE), 2.0 kg/MT betaine (BET), or 1.25 kg/MT yeast fermentate (YF) and exposed to ambient temperatures d 0–35 (CON + A, SE + A, BET + A, YF + A) or subjected to heat stress for 12 h/d on d 21–35 (CON + H, SE + H, BET + H, YF + H).

**Table 4:** Experiment 2 production outcomes of White Pekin ducks fed various dietary additives and reared under heat stress or ambient temperatures from d 0 to 35.

Treatment *	Feed Consumption (kg; d 0–35)	Feed Conversion Ratio (d 0–35)	Body Weight (kg; d 35)	Mortality (%, d 0–35)
CON + H	134.15 ^abc^	1.67	2.87 ^b^	4.17
MOS + H	131.58 ^abc^	1.68	2.80 ^b^	0.00
GT + H	129.94 ^bc^	1.65	2.84 ^b^	6.25
PS + H	128.97 ^c^	1.66	2.79 ^b^	6.25
CON + A	139.80 ^a^	1.65	3.04 ^a^	8.33
MOS + A	138.81 ^ab^	1.61	3.09 ^a^	6.25
GT + A	135.90 ^abc^	1.57	3.13 ^a^	6.25
PS + A	137.20 ^abc^	1.65	3.06 ^a^	10.42
Heat × Diet *p*-value	0.972	0.784	0.440	0.837
Main Effect Heat				
Heat Stress	131.16 ^b^	1.66	2.82 ^b^	4.17
Ambient	137.93 ^a^	1.62	3.08 ^a^	7.81
*p*-value	0.005	0.145	<0.001	0.152
Main Effect Diet				
CON	136.97	1.66	2.96	6.25
MOS	135.20	1.64	2.94	3.13
GT	132.92	1.61	2.98	6.25
PS	133.09	1.66	2.92	8.33
*p*-value	0.511	0.509	0.536	0.527
SEM	1.17	0.01	0.03	1.20

^a,b,c^ Means in the same column with different superscripts indicate a significant difference at *p* < 0.05. * Ducks were fed either a basal diet (CON) or supplemented with 0.25 kg/MT mannanoligosaccharide (MOS), 0.5 kg/MT green tea extract powder (GT), or 0.55 kg/MT Poultry Star symbiotic (PS) and exposed to ambient temperatures d 0–35 (CON + A, MOS + A, GT + A, PS + A) or subjected to heat stress for 12 h/d on d 21–35 (CON + H, MOS + H, GT + H, PS + H).

**Table 5:** Experiment 2 stress susceptibility measures and ileal histomorphology of White Pekin ducks fed various dietary additives and reared under heat stress or ambient temperatures from d 0 to 35.

Treatment *	Plasma CORT (pg/mL; d 35)	Heterophil/ Lymphocyte Ratio (d 35)	Villus Length (µm)	Crypt Depth (µm)	V/C Ratio
CON + H	7556.96 ^a^	0.60 ^ab^	708.00	150.47	5.12
MOS + H	6603.87 ^bc^	0.54 ^bc^	668.81	152.04	4.88
GT + H	4804.70 ^bc^	0.50 ^bc^	596.06	156.14	4.25
PS + H	7412.93 ^ab^	0.70 ^a^	653.88	143.35	5.01
CON + A	4380.76 ^c^	0.63 ^ab^	634.45	193.43	3.97
MOS + A	4992.40 ^abc^	0.43 ^c^	649.08	151.50	4.71
GT + A	4310.32 ^c^	0.54 ^bc^	677.45	184.56	4.33
PS + A	4454.76 ^c^	0.43 ^c^	646.46	176.89	4.11
Heat × Diet *p*-value	0.485	0.010	0.225	0.478	0.493
Main Effect Heat					
Heat Stress	6594.61 ^a^	0.59 ^a^	656.69	150.50 ^b^	4.82
Ambient	4534.56 ^b^	0.50 ^b^	651.86	176.60 ^a^	4.28
*p*-value	0.003	0.026	0.875	0.016	0.138
Main Effect Diet					
CON	5968.86	0.62	671.22	171.95	4.54
MOS	5798.13	0.48	658.95	151.77	4.80
GT	4557.51	0.52	636.76	170.35	4.29
PS	5933.84	0.56	650.17	160.12	4.56
*p*-value	0.415	0.054	0.819	0.468	0.682
SEM	353.59	0.02	13.09	5.14	0.16

^a,b,c^ Means in the same column with different superscripts indicate a significant difference at *p* < 0.05. * Ducks were fed either a basal diet (CON) or supplemented with 0.25 kg/MT mannanoligosaccharide (MOS), 0.5 kg/MT green tea extract powder (GT), or 0.55 kg/MT Poultry Star symbiotic (PS) and exposed to ambient temperatures d 0–35 (CON + A, MOS + A, GT + A, PS + A) or subjected to heat stress for 12 h/d on d 21–35 (CON + H, MOS + H, GT + H, PS + H).

**Table 6:** Experiment 3 production outcomes of White Pekin ducks fed various dietary additives and reared under heat stress or ambient temperatures from d 0 to 35.

Treatment *	Feed Consumption (kg; d 0–35)	Feed Conversion Ratio (d 0–35)	Body Weight (kg; d 35)	Mortality (%, d 0–35)
CON + H	120.27 ^bc^	1.61	2.67 ^c^	2.08
MS + H	118.00 ^c^	1.50	2.80 ^bc^	0.00
EO1 + H	117.90 ^c^	1.54	2.74 ^c^	4.17
EO2 + H	120.18 ^bc^	1.57	2.74 ^c^	0.00
CON + A	132.43 ^a^	1.57	3.01 ^ab^	2.08
MS + A	129.91 ^ab^	1.52	3.07 ^a^	2.08
EO1 + A	131.64 ^a^	1.54	3.05 ^a^	2.08
EO2 + A	132.42 ^a^	1.50	3.10 ^a^	4.17
Heat × Diet *p*-value	0.993	0.587	0.909	0.410
Main Effect Heat				
Heat Stress	119.09 ^b^	1.55	2.74 ^b^	1.56
Ambient	131.60 ^a^	1.53	3.06 ^a^	2.60
*p*-value	<0.001	0.338	<0.001	0.446
Main Effect Diet				
CON	126.35	1.59	2.84	2.08
MS	123.95	1.51	2.94	1.04
EO1	124.77	1.54	2.90	3.13
EO2	126.30	1.54	2.92	2.08
*p*-value	0.877	0.113	0.622	0.754
SEM	1.58	0.01	0.04	0.65

^a,b,c^ Means in the same column with different superscripts indicate a significant difference at *p* < 0.05. * Ducks were fed either a basal diet (CON) or supplemented with 0.09 kg/MT Essential Oil #1 (EO1), 0.5 kg/MT Essential Oil #2 (EO2), or 0.5 kg/MT Microsaf probiotic (MS) and exposed to ambient temperatures d 0–35 (CON + A, EO1 + A, EO2 + A, MS + A) or subjected to heat stress for 12 h/d on d 21–35 (CON + H, EO1 + H, EO2 + H, MS + H).

**Table 7:** Experiment 3 stress susceptibility measures and ileal histomorphology of White Pekin ducks fed various dietary additives and reared under heat stress or ambient temperatures from d 0 to 35.

Treatment *	Plasma CORT (pg/mL; d 35)	Heterophil/ Lymphocyte Ratio (d 35)	Villus Length (µm)	Crypt Depth (µm)	V/C Ratio
CON + H	12,515.3	0.48 ^b^	660.46 ^bc^	246.61 ^a^	3.81 ^b^
MS + H	10,668.2	0.62 ^ab^	784.73 ^a^	183.60 ^ab^	4.67 ^ab^
EO1 + H	12,453.4	0.82 ^a^	646.57 ^b^	142.05 ^b^	5.04 ^a^
EO2 + H	11,114.7	0.45 ^b^	666.82 ^bc^	132.47 ^b^	5.32 ^a^
CON + A	11,405.6	0.61 ^ab^	711.55 ^abc^	157.22 ^b^	4.95 ^a^
MS + A	14,621.4	0.61 ^ab^	765.92 ^ab^	185.77 ^ab^	4.93 ^a^
EO1 + A	15,700.4	0.53 ^b^	776.26 ^a^	153.45 ^b^	5.45 ^a^
EO2 + A	13,252.7	0.62 ^ab^	761.87 ^ab^	155.62 ^b^	5.35 ^a^
Heat × Diet *p*-value	0.535	0.012	0.242	0.063	0.442
Main Effect Heat					
Heat Stress	11,687.9	0.60	689.65 ^b^	176.18	4.71
Ambient	13,745.0	0.59	753.90 ^a^	163.01	5.17
*p*-value	0.118	0.977	0.018	0.421	0.069
Main Effect Diet					
CON	11,960.4	0.55	686.01	201.91 ^a^	4.38 ^b^
MS	12,644.8	0.62	775.33	184.68 ^ab^	4.80 ^ab^
EO1	14,076.9	0.67	711.41	147.75 ^b^	5.24 ^a^
EO2	12,183.7	0.54	714.35	144.04 ^b^	5.33 ^a^
*p*-value	0.657	0.262	0.123	0.034	0.032
SEM	651.92	0.03	17.80	8.74	0.15

^a,b,c^ Means in the same column with different superscripts indicate a significant difference at *p* < 0.05. * Ducks were fed either a basal diet (CON) or supplemented with 0.09 kg/MT Essential Oil #1 (EO1), 0.5 kg/MT Essential Oil #2 (EO2), or 0.5 kg/MT Microsaf probiotic (MS) and exposed to ambient temperatures d 0–35 (CON + A, EO1 + A, EO2 + A, MS + A) or subjected to heat stress for 12 h/d on d 21–35 (CON + H, EO1 + H, EO2 + H, MS + H).

## Data Availability

The original contributions presented in this study are included in the article. Further inquiries can be directed to the corresponding author.

## Abbreviations

The following abbreviations are used in this manuscript:

CORT	Plasma corticosterone
H/L	Heterophil/lymphocyte ratio
V/C	Villus/crypt ratio
BW	Body weight
FC	Feed consumption
FCR	Feed conversion ratio
CON	Control (basal diet)
BET	Betaine
EO	Essential oil
EGCG	Epigallocatechin gallate
MOS	Mannanoligosaccharide
PS	Poultry Star synbiotic
MS	Microsaf probiotic
YF	Yeast fermentate
SE	Seaweed extract
GT	Green tea
SEM	Standard error of the mean
